# Relationship between Vitamin D level and metabolic parameters in obese children and response to Vitamin D treatment

**DOI:** 10.14744/nci.2021.15870

**Published:** 2022-07-06

**Authors:** Dilek Damla Saymazlar, Ozlem Kara, Betul Biner Orhaner

**Affiliations:** 1 Department of Pediatrics, Bursa Yuksek Ihtisas Training and Research Hospital, Bursa, Turkiye; 2 Department of Pediatric Endocrinology, Bursa Yuksek Ihtisas Training and Research Hospital, Bursa, Turkiye

**Keywords:** Insulin resistance, obesity, Vitamin D deficiency

## Abstract

**OBJECTIVE:**

Vitamin D deficiency is common in children. The effects of Vitamin D on bone health are well-known. However, its effect on glucose and lipid metabolism in obese children remains controversial. This study projected to evaluate the association between Vitamin D level and glucose, lipid, and bone metabolism parameters in obese children. In addition, the objective of the study was to determine the change in insulin resistance after Vitamin D replacement therapy in obese children with Vitamin D deficiency.

**METHODS:**

Hundred fifty children with obesity were included in our retrospective cross-sectional study. The patients were separated into two groups as the study group (serum 25(OH)D level <20 ng/ml) and the control group (serum 25(OH)D level ≥20 ng/ml). Physical examination, body fat mass, and laboratory findings of the two groups were compared. Moreover, patients in the study group were supplemented with Vitamin D 2000 IU/d for 24 weeks. Glucose, insulin levels were analyzed before and after treatment.

**RESULTS:**

Body fat mass and percentage were evaluated as more raised in the study group than those in the control group. The study group had a higher level of insulin resistance. There was a significant loss in body weight of patients after treatment in the study group and insulin resistance of the study group decreased after Vitamin D3 treatment.

**CONCLUSION:**

Considering the low side effects and affordability of Vitamin D, it would be a reasonable approach to identify serum Vitamin D levels in obese children and to administer a treatment to those with Vitamin D deficiency.

## Highlight key points

- Vitamin D deficiency and obesity are common health problems.
- Vitamin D deficiency is a common condition in obese children.
- In recent years, Vitamin D receptor is detected in many organ groups other than bone, kidney, and intestine and this reveals the presence of other systemic effects of Vitamin D.
- The body mass index of children with Vitamin D deficiency was higher.
- Obese children with Vitamin D deficiency had a higher insulin resistance.
- Obese children had a significant decrease in insulin resistance after the Vitamin D treatment, together with appropriate nutrition and exercise schedule.

Obesity rates in childhood are gradually increasing in the world and it is becoming a health problem [1]. Recent studies shows that Vitamin D deficiency plays a crucial role in the development of obesity [2]. Besides, Vitamin D deficiency is frequently seen, especially in obese people [3]. This is explained by many factors as follows; accumulation of vitamin dissolved in fat due to increased adipose tissue in obese people, insufficient benefiting from sunlight as a result of a sedentary lifestyle, volumetric dilution due to increased bone size [4]. Vitamin D is not only effective in the musculoskeletal system; furthermore, it is a hormone with receptors in every cell and tissue in our body [5]. It is a known fact that obesity causes entities such as Type II diabetes, dyslipidemia, and hypertension in children [6]. However, in recent years, Vitamin D and its effect on the development of obesity-related complications are significantly interested [6, 7]. Also, many studies focus on Vitamin D supplementation and change in metabolic parameter in obese adults [8]. These studies are limited to obese children [6, 9].

In this study, we decided to evaluate the association between Vitamin D level and body fat mass, glucose, lipid, and bone metabolism parameters in obese children and adolescents and to identify the change in insulin resistance before and after Vitamin D treatment in obese patients with Vitamin D deficiency.

## MATERIALS AND METHODS

### Study Population

Patients aged between 7 and 18 years, who had been admitted to the pediatric endocrinology outpatient clinic with the complaint of being overweight and whose body mass index (BMI) >95^th^ percentile for age and gender were included in the study. The study protocol was approved by the Bursa Yuksek Ihtisas Training and Research Hospital Clinical Research Ethics Committee (2011-KAEK-25 2019/02-09) under the principles of the Declaration of Helsinki. Patients with syndromic and secondary obese, those with chronic diseases, and those receiving medications and vitamin supplements were excluded from the study. Body weight and height, BMI, body-fat measurement, physical examination findings (puberty status, stria, acanthosis nigricans, and blood pressure), laboratory findings (blood glucose, serum insulin, and serum total 25-hydroxyvitamin D (25(OH)D), calcium (Ca), phosphor (P), alkaline phosphatase (ALP), and parathyroid hormone (PTH) level of the patients were recorded in the patient files. Serum 25(OH) D level of <20 ng/ml was evaluated as a Vitamin D deficiency [10]. Patients with a similar BMI were separated into two groups according to 25(OH)D level. The study group included patients with serum 25(OH)D level of <20 ng/ml and the control group included those with serum 25(OH)D level ≥20 ng/ml. The groups were compared in terms of physical examination and laboratory findings. Furthermore, patients in the study group received Vitamin D3 (cholecalciferol) 2000 IU/d treatment for 24 weeks along with adequate nutrition and exercise advice [11]. Changes in body weight, blood glucose, insulin levels, and homeostasis model assessment for insulin resistance (HOMA-IR) index were compared before and after the treatment.

### Anthropometric Measurements

Body fat analysis of all patients admitted to the pediatric endocrinology clinic presenting with obesity is performed using Tanita Body Composition Analyzer SC-330 equipment. The operation principle of the equipment is based on Bioimpedance analysis. Body analysis is performed by giving 50 kHz electric current to the body through the foot electrode.

### Laboratory Measurements

Serum fasting blood glucose, lipid profile (total cholesterol, triglyceride, high-density lipoprotein cholesterol [HDL-C], and low-density lipoprotein cholesterol [LDL-C]), and insulin levels were tested after 12 h of fasting. HDL-C levels of <35 mg/dl, LDL-C levels of >130 mg/dl, and triglyceride level of >150 mg/dl were considered dyslipidemia. Vitamin D insufficiency was defined as a 25(OH)D level of <20 ng/ml and a 25(OH)D level of ≥20 ng/ml was considered as sufficient Vitamin D status [10]. Insulin resistance was calculated with HOMA-IR formula (fasting blood glucose (mg/dl) × fasting insulin (µIU/ml)/405).

### Treatment Protocol

All patients diagnosed with obesity received adequate nutrition and exercise advice. Furthermore, patients in the study group received oral Vitamin D3 (cholecalciferol) 2000 IU/d treatment for 24 weeks [11]. Those patients were quarterly evaluated in terms of physical examination and laboratory findings. After 24 weeks, levels of fasting glucose, fasting insulin, and HOMA-IR were compared to pre-treatment levels.

### Statistical Analysis

Statistical analysis of data was performed using SPSS 22.0 IBM Corp., USA, Ill statistics package software. The distribution of variables was evaluated with a Kolmogorov–Smirnov test. In descriptive statistics of the data, mean±standart deviation for normally distributed variables and median (25–75%) values for non-normally distributed variables were used. According to their distribution, unpaired t-test and Mann–Whitney U test were applied. Within the study group, changes in laboratory measurements (baseline vs. 24 weeks) were evaluated using the Wilcoxon signed-rank test and paired t-test. Considering the association between obesity and puberty, all comparisons of variables between two groups were adjusted for gender and puberty using ANCOVA for normally distributed data and ranked ANCOVA for non-normally distributed data. Qualitative data were evaluated using the chi-square test. P<0.05 was accepted as a cutoff value for statistical significance.

## RESULTS

The files of all obese patients admitted to the pediatric endocrinology outpatient clinic between January 1 and September 1, 2018, were analyzed. A total of 150 obese patients were involved in this study, whose physical examination, laboratory findings on admission were accessible. The patients were separated into two groups, first group being detected as serum 25(OH)D level ≥20 ng/ml (control group) and second group being detected as serum 25(OH)D level <20 ng/ml (study group). The mean age was 13.1±3.07 years and the age distribution of the two groups was similar. However, when the groups are evaluated according to pubertal stage, there were more pubertal patients in the study group (p=0.013). Although there was no statistical difference in body weight, body height, and BMI measurements in both groups; when the effects of gender and puberty were adjusted, the BMI level of the study group was found to be higher (adj. p=0.014). Body fat mass and percentage levels were remarkably higher in the study group (p<0.001). It was also found statistically significant when the effects of gender and puberty were adjusted.

Values of fasting glucose, fasting insulin, and HOMA-IR were higher in the study group than in the control group (respectively p=0.003, <0.001, and <0.001). The serum lipid profile was similar in both groups (p>0.05). However, when the effects of gender and puberty were adjusted, HDL, LDL, and TC levels were higher in the control group.

Serum Ca, P, and ALP values, which are within the bone metabolism parameters, were evaluated as low in the study group (p<0.001). PTH level was measured more raised in the study group (p=0.02). In addition, the difference in these laboratory findings was found statistically significant once the effects of gender and puberty were adjusted. None of the patients had a history of fracture. Demographic, physical, clinical findings, and laboratory variables are shown in Table 1.

**Table 1:** Comparison of demographics, clinical, physical findings, and laboratory variables between two group

	Study group (n=75)	Control group (n=75)	p	Adj. P
Age, years	13.41±2.67	12.82±3.42	0.285^¶^	
Gender (female) %	65	55	0.182^‡^	
State of puberty (pubertal) %	67	47	**0.013** ^‡^	
Presence of acanthosis nigricans %	53	47	0.414^‡^	
Blood pressure (hypertensive) %	3	3	1.00^‡^	
History of bone fracture	-	-	1.00^‡^	
Body weight (kg)	72.90±17.26	66.42±21.33	0.060^¶^	0.060
Body weight sd	2.93±0.88	2.74±0.95	0.115^¶^	**0.041**
Height (cm)	157.11±13.34	154.38±13.18	0.211^¶^	**0.035**
Height sd	1.08±0.80	1.27±0.81	0.169^£^	0.704
BMI (kg/m2)	30.28±4.11	29.38±3.91	0.275^¶^	**0.014**
BMI sd	2.65±0.63	2.49±0.67	0.152^£^	0.521
Body fat percentage (%)	36.26±5.59	29.56±9.92	<**0.001**^¶^	**0.009**
Body fat mass (kg)	28.09±9.37	20.34±10.62	<**0.001**^¶^	**0.008**
25(OH)D, ng/ml	11.68±4.45	27.97±4.94	<**0.001**^£^	<**0.001**
Glucose, mg/dl	91±11.7	86.5±7.1	**0.003** ^£^	**0.001**
Insulin, µIU/ml	26.18±25.64	15.83±10.10	<**0.001**^¶^	**0.015**
HOMA-IR	6.07±6.62	3.31±2.12	<**0.001**^¶^	**0.016**
Triglycerides, mg/dl	106.54±48.52	101.72±39.61	0.880^¶^	0.304
HDL-C, mg/dl	46.85±9.04	47.28±8.88	0.749^¶^	**0.031**
LDL-C, mg/dl	95.62±31.42	102.23±29.33	0.125^¶^	**0.004**
TC, mg/dl	163.34±34.10	168.92±31.47	0.162^¶^	**0.004**
Calcium, mg/dl	9.92±0.45	10.09±0.25	<**0.001**^¶^	**0.004**
Phosphorus, mg/dl	4.34±0.58	4.82±0.62	<**0.001**^¶^	**0.004**
ALP, IU/L	146.68±75.34	198.16±93.26	**0.001** ^¶^	**0.006**
PTH, pg/ml	48.02±12.21	43.20±18.66	**0.024** ^¶^	**0.004**

Descriptive statistics were given as mean±sd or n (%). Adj. p, P-values obtained by adjusted to puberty and gender. ¶: Mann–Whitney U test; £: Independent samples t-test^¶^; ‡: Chi-square test; BMI: Body mass index; ALP: Alkaline phosphatase; HOMA-IR: Homeostasis model assessment for insulin resistance; HDL-C: High-density lipoprotein cholesterol; IQR: 25^th^ and 75^th^ percentiles; LDL-C: Low-density lipoprotein cholesterol; PTH: Parathyroid hormone; SD: Standard deviation; TC: Total cholesterol.

All obese patients received adequate nutrition and exercise schedule. Patients with Vitamin D deficiency were administered oral Vitamin D3 2000 IU/d treatment. After 24 weeks of treatment, changes in body weight, total 25(OH)D, glucose, insulin, and HOMA-IR levels were evaluated before and after treatment. There was a significant weight loss after 24 weeks (p<0.001). Moreover, there was notable decrease in blood glucose, fasting insulin, and HOMA-IR levels (respectively, p=0.004, <0.001, and <0.001). The before and after treatment comparison is shown in Table 2.

**Table 2:** Comparison of weight and laboratory variables within study group

Study group	Pretreatment		After treatment		p
	Mean±SD	Median (IQR)	Mean±SD	Median (IQR)	
Weight, kg	72.99±17.26	72.7 (60.9–85.5)	67.19±15.94	68.0 (58.1–80.4)	<**0.001**^¥^
Weight, sd	2.93±0.88	2.62 (2.34–3.45)	2.58±0.86	2.40 (1.90–3.0)	<**0.001**^β^
25(OH)D, ng/ml	11.68±4.45	11.9 (7.4–15.2)	21.05±1.52	20.3 (20–21)	<**0.001**^β^
Glucose, mg/dl	91.08±11.69	90 (85–97)	86.04±7.26	87 (81–91)	**0.004** ^β^
Insulin, µIU/ml	26.18±25.64	20.6 (14.3–28.6)	21.25±15.50	18.6 (11.9–23.7)	<**0.001**^β^
HOMA-IR	6.07±6.62	4.3 (2.9–6.5)	4.22±2.64	3.9 (2.4–5.0)	<**0.001**^β^

SD: Standard deviation; HOMA-IR: Homeostasis model assessment for insulin resistance; IQR: 25^th^ and 75^th^ percentiles; ¥: Paired samples t-test; β: Wilcoxon signed-rank test.

## DISCUSSION

In this article, the association between Vitamin D level and metabolic parameters in obese children was evaluated. Body fat mass and percentage, fasting blood glucose, fasting insulin, and HOMA-IR values were detected to be raised in the study group (serum 25(OH)D level <20 ng/ml). Furthermore, a decrease in glucose, insulin, and HOMA-IR levels was identified after Vitamin D3 supplementation of patients with Vitamin D deficiency.

Vitamin D deficiency and obesity are frequent diseases almost all over the world. This might be coincidental, but the identification of a low level of Vitamin D in obese people in many studies indicates that there may be an important relationship [2, 3, 12]. There are many studies evaluating Vitamin D levels and BMI. Greene et al. [12] in their research using Canadian health measurement survey data indicated that overweight and obese children had a lower level of Vitamin D compared to children with normal BMI. In addition, this study found an inverse association between 25OH Vitamin D level and body fat percentage. In studies where an inverse association between Vitamin D level and body fat mass amount was identified, body fat mass amount was evaluated not by any measurement, but by accepting BMI and body fat amount as the same. In our study, moreover, we measured body fat mass amount of patients included in the study through Tanita Body Composition Analyzer SC-330, which is a kit for the analysis of body fat. There was higher body fat mass and percentage in the group with Vitamin D deficiency (p<0.001).

During the recent years, it is broadly studied that Vitamin D and its effects on the musculoskeletal system, developing insulin resistance, diabetes, and cardiovascular diseases, especially in obese patients [6, 7, 13, 14]. Many studies focus on the link between Vitamin D and glucose homeostasis. An in vitro animal study marked that insulin secretion was altered in rats without a functional Vitamin D receptor and that there was a significant elevation in insulin secretion from the pancreas after application of Vitamin D supplementation [15]. Accordingly, many clinical studies have been assessed. In their meta-analysis study, Pittas et al. [16] demonstrated that there was an inverse association between insulin resistance and 25OH Vitamin D. Boucher et al. [17] indicated in their epidemiological study that the serum Vitamin D level was lower in patients with Type II diabetes developing risk factors than in patients without having Type II diabetes developing risk factors. Our study marked that the group with Vitamin D deficiency had higher levels of glucose, insulin, and HOMA-IR compared to the group with sufficient Vitamin D level (p<0.001).

The effects of Vitamin D on bone health are well-known. Even though obesity is considered as have positive effects on bone mineral density (BMD), studies conducted in recent years have supported the opposite [18]. Although BMD is high in obese people, the opinion prevails about the greater fragility of bone tissue and the decrease in bone resistance. In our study, we identified that serum Ca and P levels in the group with Vitamin D deficiency were lower than those in the control group, though they were in the normal range. Similarly, PTH levels were also elevated. However, there was no history of fracture in patients. There are many studies analyzing especially the relationship between BMI and bone fracture and osteoporosis in adults, but it is limited in children [18, 19]. The most important complication related to obesity researched recently is a metabolic syndrome, which is evaluated to be the reason for severe morbidity and mortality. In their meta-analysis study, Khan et al. [20] indicated that there was a significant association between Vitamin D level and Type II diabetes, metabolic syndrome, and insulin resistance. Pittas et al. [16] also demonstrated in their meta-analysis study that there was a link between Vitamin D level and Type II diabetes, metabolic syndrome, and insulin resistance. In our study, we did not evaluate the metabolic syndrome criteria, but HDL, LDL, and total cholesterol levels which are the parameters of dyslipidemia were more raised in the control group, though they were in the normal range.

In the lower numbers of studies, differences in fasting blood glucose, insulin, and HOMA-IR levels before and after Vitamin D treatment in obese children were evaluated and there was no specific consensus on the dose of treatment [9, 21]. In our study, we dosed Vitamin D (cholecalciferol) 2000 IU/d treatment to our patients in the study group. We re-evaluated the body weight and laboratory data of these patients from the patient files after 24 weeks of treatment. There was a remarkable increase in 25(OH)D levels after the treatment (p<0.001). In their study, Castaneda et al. [21] administered Vitamin D3 (cholecalciferol) 2000 IU/d treatment for 12 weeks to obese and non-obese adolescents. After the treatment, there was no evident elevation in 25(OH)D levels of obese patients. Eventually, it was demonstrated that the administration of a higher dose of Vitamin D treatment was required in obese patients.

After 24 weeks of treatment, changes in glucose homeostasis of treated patients were evaluated from patient files and there were significant decreases in glucose, insulin, and HOMA-IR levels. In limited studies analyzing the response to Vitamin D treatment in obese children, there were different results. Milajerdi et al. [22] indicated in their meta-analysis study that Vitamin D supplements in patients with chronic renal failure had a positive impact on glucose, HOMA-IR, triglyceride, and cholesterol levels, but had not affected on insulin, HbA1c, LDL, and HDL cholesterol. In their randomized, controlled study Belenchia et al. [9] administered a placebo and Vitamin D3 4000 IU/d treatment to obese adolescents and demonstrated no difference in BMI and glucose level after 6 months. In another study, there was no change in lipid profile and insulin resistance [6]. We cannot attribute the discernible improvement in glucose homeostasis in our study solely to Vitamin D3 treatment. The reason is that together with Vitamin D3 treatment, patients adjusting to adequate nutrition and exercise schedule had a significant weight loss after 24 weeks (p<0.001).

The most important limitation of our study is that it is a retrospective study. Patient data were evaluated from patient files. Furthermore, as there was no control group in the evaluation of treatment response, it caused a limitation for the assessment of the efficacy of Vitamin D3 treatment. In addition, another limitation of the study was that the effects of diet, exercise, and Vitamin D treatment on weight loss were not evaluated as separate parameters since it was a retrospective study.

By way of conclusion, Vitamin D deficiency has negative impact on glucose, insulin, and HOMA-IR levels in obese children and adolescents. Meanwhile, an increase in body fat mass was identified in obese people with Vitamin D deficiency. Even though they were in the normal range, there was a decrease in serum Ca and P levels, and an increase in serum PTH level. There was no history of fracture in patients. There was a significant weight loss after Vitamin D3 supplementation of patients with Vitamin D deficiency for 24 weeks. In addition, a decrease in glucose, insulin, and HOMA-IR levels was identified. In conclusion, when considering the low cost and low side effect incidence of Vitamin D supplementation and replacement therapy, along with adequate nutrition and exercise schedule in obese patients, the identification of serum Vitamin D levels and the administration of treatment in those with deficiency will be a reasonable approach and could be concluded as a cost effective therapy as well. Further prospective and long-term researches in this field would be of great to confirm the results of our study.
